# Disruption of Functional Membrane Microdomains Enhances Methicillin-Resistant *Staphylococcus aureus* Pathogenesis via Hyperexpression of Hemolysins

**DOI:** 10.3390/vetsci13080839

**Published:** 2026-08-20

**Authors:** Bingtian Jin, Changzhen Wang, Tiantian Liu, Pengcheng Dong, Xurong Wang, Xiao Yang, Dengwang Yuan, Feng Yang

**Affiliations:** 1 Lanzhou Institute of Husbandry and Pharmaceutical Sciences, Chinese Academy of Agricultural Sciences, Lanzhou 730050, China; xj92624@163.com (B.J.); wangchangzhen@caas.cn (C.W.); 15848156806@163.com (T.L.); dongpengcheng@caas.cn (P.D.); wangxurong@caas.cn (X.W.); yangxiao02@caas.cn (X.Y.); 19556907626@163.com (D.Y.); 2 The First School of Clinical Medicine, Gansu University of Chinese Medicine, Lanzhou 730000, China; 3 Key Laboratory of Veterinary Drug Discovery, Ministry of Agriculture and Rural Affairs, Lanzhou 730050, China

**Keywords:** functional membrane microdomains, two-component system, methicillin-resistant *Staphylococcus aureus*, hemolysis

## Abstract

(1) Background: methicillin‑resistant *Staphylococcus aureus* (MRSA) is a zoonotic pathogen, and its hemolysins serve as key virulence factors. Functional membrane microdomains (FMMs) are protein-enriching platforms and regulate diverse physiological functions by recruiting and assembling various proteins. However, whether and how FMMs regulate the hemolytic ability of MRSA remains unclear. This study aimed to investigate FMM-mediated regulation of MRSA hemolysins and the underlying mechanisms. (2) Methods: Homologous recombination was employed to generate FMM-disrupted (N315Δ*floA*) and complemented (N315Δ*floA*::*floA*) strains from the MRSA N315 wild-type strain (N315 WT). The three strains were compared with respect to hemolytic activity, transcript levels of key virulence and regulatory genes, and in vivo virulence. (3) Results: Disruption of FMMs significantly enhanced hemolytic activity compared with N315 WT and complemented strains. Meanwhile, FMM disruption repressed the two-component system genes (*vraS* and *vraR*), while activating the *agr* operon (*agrB*, *agrD*, *agrC* and *agrA*) and its effector molecule RNAIII, leading to upregulation of hemolysin genes (*hla*, *hlb*, *hld*). In vivo, N315Δ*floA* infection markedly increased mortality in *G. mellonella* larvae and BALB/c mice, with significantly elevated pro-inflammatory factors (TNF-α, IL-6, and IL-1β) in mouse plasma. All these phenotypes were effectively reversed in N315Δ*floA*::*floA*. (4) Conclusions: Disruption of FMMs potentiates both hemolytic activity and overall virulence in MRSA, with the potential underlying mechanism involving the VraS/R-Agr regulatory axis that drives transcriptional upregulation of hemolysin-encoding genes.

## Simple Summary

The high pathogenicity of methicillin-resistant *Staphylococcus aureus* (MRSA) is mainly attributed to elevated hemolysin expression. The current study explored the effects of functional membrane microdomains (FMMs) on hemolysin expression and virulence in MRSA, aiming to find new clues for the prevention and treatment of MRSA infections. Our findings demonstrate that FMMs function as a negative regulator of MRSA virulence. FMMs repress hemolysin expression and excessive host inflammatory responses through modulating the VraS/R-Agr signaling axis. This provides novel mechanistic insights into FMM-mediated regulation of hemolysis and offers a theoretical basis for developing anti-MRSA therapeutic strategies targeting FMMs.

## 1. Introduction

Methicillin-resistant *Staphylococcus aureus* (MRSA) remains one of the most formidable pathogens of the 21st century, posing a persistent threat to global public health [1]. The hallmark of MRSA is its resistance to most β-lactam antibiotics and multiple other antimicrobial classes currently available for therapy [2]. Beyond antimicrobial resistance, MRSA harbors a diverse array of virulence factors, including adhesins, hemolysins, biofilm-associated factors, and immune modulators [3,4], facilitating the bacteria to avoid the immune system and contribute to increased severity of infections [5]. Hemolysins (α, β, γ, and δ) are the most important exotoxins that damage erythrocytes and platelets, destroy lysosomes, and usually cause local ischemia and necrosis [6].

In *S. aureus*, hemolysin expression is tightly regulated by an elaborate transcriptional network, with the accessory gene regulator (Agr) quorum sensing (QS) system serving as the central coordinator [7]. As a cell density-dependent signaling pathway [8], the agr operon contains divergent promoters P2 and P3 that transcribe transcripts RNAII (encoding AgrB, AgrD, AgrC, and AgrA) and RNAIII, respectively [9,10]. During the regulatory process, the P2 promoter drives the autoregulation of the *agrBDCA* operon, sustaining continuous QS activation. Specifically, *S. aureus* generates a precursor peptide via AgrD. The peptide is processed by the peptidase AgrB to form an auto-inducing peptide (AIP) [11]. Once secreted extracellularly and reaching a critical threshold, AIP binds to receptor AgrC and triggers autophosphorylation of its histidine kinase domain. The phosphate group is subsequently transferred to AgrA, thereby activating the P2 and P3 promoters within the agr operon [12,13]. RNAIII transcribed from the activated P3 promoter regulates the expression of various virulence factors in *S. aureus*, including α-hemolysin (Hla), β-hemolysin (Hlb), δ-haemolysin (Hld), biofilm formation, and toxic shock syndrome toxin-1 (TSST-1) [14].

Agr system activation is finely tuned by environmental signals transduced through two-component systems (TCSs), each typically composed of a membrane-bound sensor histidine kinase (HK) and a cytoplasmic response regulator (RR) [15]. Signal transduction and the regulation of downstream genes are achieved through phosphate group transfer between proteins [16]. Notably, VraS/R contributes to hemolysin gene expression in *S. aureus* by cross-regulating with the Agr system. This cross-talk highlights that hemolysin regulation is a multi-layered process, integrating the Agr and TCSs to fine-tune MRSA pathogenicity [17,18,19].

Functional membrane microdomains (FMMs) are structurally organized microdomains enriched in scaffold protein flotillins and polyisoprenoid compounds. The FMM marker flotillins are responsible for recruiting proteins to the FMMs [20,21], which serve as platforms for the enrichment of various membrane proteins and the assembly of protein complexes, participating in essential biological processes such as signal transduction, substance transport, and pathogen recognition [22,23]. Disruption of FMMs via knockout of flotillin-encoding gene *floA* in MRSA affects bacterial virulence, antibiotic resistance, biofilm formation, motility, and membrane fluidity [24,25,26,27,28]. However, the specific role of FMMs in hemolysin regulation, particularly its impact on MRSA hemolysin expression and their interplay with the Agr system and TCSs, remains poorly defined.

In this study, we constructed FMM-disrupted (N315Δ*floA*) and complemented (N315Δ*floA*::*floA*) strains from the MRSA N315 wild-type strain to investigate the following objectives: (1) to determine whether FMMs regulate the hemolytic activity of MRSA; (2) to elucidate the molecular mechanism by which FMMs modulate hemolysin expression, with particular focus on the VraS/R-Agr signaling axis; and (3) to evaluate the impact of FMM disruption on in vivo virulence using both invertebrate (*G. mellonella*) and murine infection models. These findings offer new insights into FMM-targeted anti-MRSA strategies and a theoretical basis for developing novel anti-virulence therapeutics.

## 2. Materials and Methods

### 2.1. Bacterial Strains, Plasmids, and Growth Conditions

The bacterial strains and plasmids used in this study are listed in Table 1. The standard MRSA N315 strain, *S. aureus* RN4220 strain, the temperature-sensitive shuttle vector plasmid pBT2, and the complementation plasmid pLI50 were all kindly provided by Prof. Xiancai Rao (Army Medical University, China). The *Escherichia coli* strain DH5α was purchased from Shanghai Weidi Biotechnology Co., Ltd. (Shanghai, China). *E. coli* strains were cultivated with shaking (220 rpm) in Luria–Bertani (LB) medium (HuanKai Microbial, Guangzhou, China) or on Luria–Bertani agar (LA) at 37 °C. *S. aureus* strains were grown with shaking (220 rpm) in Tryptic Soy Broth (TSB) medium (Oxoid, Basingstoke, UK), Brain Heart Infusion (BHI) medium (Oxoid, Basingstoke, UK), or on Tryptic Soy Agar (TSA) at 37 °C. When needed, the agar plates or broth media were supplemented with chloramphenicol (Cm, 20 μg/mL) or ampicillin (Amp, 100 μg/mL) to culture strains carrying the corresponding plasmids.

### 2.2. Preparation of Electrocompetent S. aureus Cells

Preparation of electrocompetent *S. aureus* cells was performed following previously described protocols with minor modifications [29]. *S. aureus* cells were streaked from −80 °C storage onto TSA plates and incubated at 37 °C. A single colony was picked and inoculated into 2 mL TSB for overnight incubation at 37 °C. 1 mL overnight culture was added to 100 mL BHI in a 250 mL conical flask, and shaken at 37 °C until an OD_600_ of 0.5 was reached. The culture was put on ice for 30 min and then transferred to a sterile conical-bottom centrifuge tube. The cells were collected by centrifugation at 5000 rpm at 4 °C for 10 min; the supernatant was then discarded, and the cell pellet was gently resuspended in 30 mL of ice-cold 0.5 M sucrose. The suspension was kept on ice for 10 min. This centrifugation and resuspension were repeated three times. Finally, the cells were resuspended in 600 μL ice-cold 0.5 M sucrose and stored at −80 °C.

### 2.3. Plasmid Extraction and Transformation in S. aureus

Plasmids from all *S. aureus* strains were isolated using a plasmid miniprep kit (Omega Bio-Tek, Norcross, GA, USA) according to the manufacturer’s instructions, except that the cells were pretreated with digestion buffer containing 40 U/mL lysostaphin and10 mg/mL lysozyme, and 10% (v/v) glycerol for 60 min. Plasmids were transformed into all *S. aureus* strains by electroporation. Homologous recombination vector DNA (100–500 ng) and electrocompetent *S. aureus* cells (100 μL) were mixed and placed in a Gene Pulser cuvette with a 0.2 cm electrode gap, and the cuvette was put on ice for about 30 min. The settings for electroporation were as follows: Voltage, 2.5 kV; Capacitor, 50 μF; Resistance, 200 Ω. After electroporation, 700 μL TSB was added to the cuvette immediately, and the cuvette was kept on ice for about 10 min. Then the cells were transferred into a 1.5 mL microcentrifuge tube and incubated with shaking (150 rpm, 30 °C) for 1 h before being spread onto a TSA plate (Cm, 20 μg/mL).

### 2.4. Construction of the FMM-Disrupted Strain

To induce deletion of the *floA* gene in *S*. *aureus* N315, allelic replacement was applied. Plasmid pBT2 was utilized to construct the FMM-disrupted strain (N315Δ*floA*). Fusion fragments (pUC57-Δ*floA*) containing the upstream and downstream homologous arms of the target *floA* gene were designed and synthesized based on the genomic sequence of *S. aureus* strain N315 (GenBank accession number: BA000018.3). The resulting fragments were digested with *EcoR* I and *Kpn* I and then cloned into pBT2. Afterward, the resulting plasmid pBT2-Δ*floA* was electroporated into *S. aureus* strain RN4220 for modification and then transferred into *S. aureus* strain N315. Finally, an allelic replacement mutant was selected: The N315 strain carrying the homologous recombination vector was inoculated at a 1:1000 dilution into 2 mL of TSB (Cm, 20 μg/mL) medium and incubated with shaking (30 °C, 200 rpm) for 12 h. The next day, the culture was transferred at a 1:100 ratio into 2 mL TSB medium and incubated with shaking (42 °C, 200 rpm), with subculturing every 24 h for a total of 5 passages. The culture was streaked onto TSA plates (Cm, 20 μg/mL) and incubated at 42 °C for 12 h. On the following day, a single colony was picked and transferred into 2 mL of TSB medium and incubated with shaking (30 °C, 200 rpm), with subculturing every 24 h for a total of 3 passages. The culture was streaked onto TSA plates and incubated at 30 °C for 12 h to obtain purified single colonies. Single colonies were randomly picked and transferred into a 96-well plate containing 200 μL TSB medium and cultured at 30 °C until turbid; the cultures were then transferred from the 96-well plate at a 1:100 ratio into another 96-well plate containing TSB medium (Cm, 20 μg/mL) and incubated at 30 °C for 12 h. Clones that grew in plain TSB medium but not in Cm-containing TSB medium were selected by comparing the two 96-well plates. Successful deletion of the target *floA* gene was confirmed by PCR and first-generation Sanger DNA sequencing. Primers are listed in Table 2.

### 2.5. Construction of the FMM-Complemented Strain

To complement the *floA* gene and restore FMM function in the FMM-disrupted strain, the genome of *S. aureus* N315 (GenBank accession number: BA000018.3) was used as the template, and a pUC57-*floA* fusion plasmid containing the open reading frame (ORF) of the *floA* gene and its promoter region was designed and synthesized. The fusion fragment was digested with *EcoR* I and *BamH* I, then cloned into the pLI50 vector. Next, the resulting plasmid pLI50-*floA* was first electroporated into RN4220 for modification, and then transformed into MRSA N315Δ*floA* to generate the FMM-complemented strain (N315Δ*floA*::*floA*). The correct integration of the wild-type *floA* gene into N315ΔfloA was verified by PCR amplification using primers *floA*-F/*floA*-R, followed by Sanger sequencing of the entire *floA* open reading frame and its promoter region to confirm the absence of any mutations.

### 2.6. Growth Curve Measurement

To investigate whether FMMs affect the growth of *S*. *aureus* N315, the overnight cultures of N315 wild-type (WT), N315Δ*floA*, and N315Δ*floA*::*floA* were first measured for their OD_600_ values and adjusted to the same level. Then these cultures were inoculated into equal volumes of TSB liquid medium at a volume ratio of 1:100, followed by incubation with shaking at 37 °C, 220 rpm. The OD_600_ values were measured every 1 h using a microplate reader (Multiskan FC; Thermo Fisher Scientific, Waltham, MA, USA) until the bacteria reached the stationary phase. Finally, bacterial growth curves were plotted with OD_600_ values as the ordinate and time as the abscissa, and performed in triplicate.

### 2.7. Hemolysis Assay

To assess the hemolytic activity of the target bacterial strain, *S. aureus* RN4220, which only expresses β-hemolysin and exhibits typical hot-cold hemolysis characteristics, was selected as the positive control [30,31]. Overnight cultures of N315 WT, N315Δ*floA*, N315Δ*floA*::*floA*, and RN4220 were diluted 1:1000 with PBS. Then, 0.5 μL of the bacterial suspension was spotted onto Columbia sheep blood agar plates (HuanKai Microbial, Guangzhou, China). After incubating at 37 °C for 12 h, the hemolysis zones were observed and photographed. The plates were then sealed and placed at 4 °C for 12 h to stand, after which observations and photographs were taken again. The diameter of the hemolysis zone was measured using Adobe Photoshop CC 2023 software to quantitatively analyze the hemolytic activity of the strains.

According to the mechanism of hemolysin interaction, α-hemolysin (Hla), β-hemolysin (Hlb), and δ-hemolysin (Hld) were qualitatively detected using the cross-streaking method [32]. RN4220 was used as a reference for vertical cross-streaking, and the degree of hemolysis at the cross indicates phenotypes of different hemolysins: the same as for RN4220 is determined as β-hemolysin, the attenuated hemolysis is determined as α-hemolysin, and the enhanced hemolysis is determined as δ-hemolysin.

β-hemolysin production was further verified by CAMP test [33]: *Streptococcus agalactiae* ATCC12386 was streaked at the center of a blood agar plate, and the test strains were streaked perpendicularly at a distance of 0.5–1.0 cm. The plate was incubated at 37 °C for 24 h. The appearance of an arrow-shaped hemolytic zone at the junction indicates a positive result for β-hemolysin; the absence of this feature indicates a negative result. RN4220 was used as the positive control in the experiment.

### 2.8. RT-qPCR

To compare the transcript levels of hemolysins and regulatory genes among N315 WT, N315Δ*floA*, and N315Δ*floA*::*floA*, we performed RT-qPCR to examine the expression of the co-transcribed agr operon (*agrB*, *agrD*, *agrC*, *agrA*) and its effector molecule RNAIII, the hemolysin genes *hla*, *hlb*, *hld*, and the genes *vraS* and *vraR* of the two-component system VraS/R. Notably, in the N315 strain used in this study, the *hlb* locus is truncated by a prophage insertion, generating two separate fragments (SA1752 and SA1811) [34]. *S. aureus* strains of interest at the logarithmic phase were prepared. Total RNA and the relevant cDNA were obtained using the SteadyPure Universal RNA Extraction Kit and the Evo M-MLV RT Mix Kit with gDNA Clean for qPCR Ver.2 (Accurate Biotechnology (Hunan) Co., Ltd., Changsha, China), respectively. Quantitative polymerase chain reaction (qPCR) was performed, and the relative expression level of each gene was calculated as a ratio of its threshold cycle (Ct value) relative to that of the reference gene *hu* [18,35]. The primers used are listed in Table 2. Relative gene expression levels were determined using the 2^−ΔΔCt^ method [36].

### 2.9. Galleria Mellonella Infection Model

The invertebrate greater wax moth *G*. *mellonella* was used as an in vivo model to examine the pathogenicity of diverse *S. aureus* strains. Infection experiments were performed according to the protocol described by Xiao et al. [37]. Each larva was gently injected with 10 μL of bacterial suspension (1 × 10^8^ CFU/mL) into the left hind proleg, and larvae injected with 10 μL of PBS served as the control group. After injection, the larvae were placed in clean Petri dishes and incubated at 37 °C to allow infections to progress for 96 h. During incubation, larval mortality was recorded every 12 h (larvae were considered dead when unresponsive to gentle tapping). Three independent experiments were performed, with 15 larvae per strain and per experiment. Survival curves were constructed using the Kaplan–Meier method.

### 2.10. Murine Infection Model

A mouse intraperitoneal infection model was adopted to compare the in vivo pathogenic potential of *S. aureus* N315 WT, N315Δ*floA* and N315Δ*floA*::*floA*. Six-week-old female BALB/c mice (SPF) were obtained from the Laboratory Animals Center of Lanzhou Veterinary Research Institute, Chinese Academy of Agricultural Sciences. The mice were housed in polypropylene cages in standardized lighting conditions and had *ad libitum* access to food and water. All animal procedures were performed in accordance with the Animal Ethics Committee of the Lanzhou Institute of Husbandry and Pharmaceutical Sciences and the Chinese Academy of Agricultural Sciences. Mice were randomly assigned to experimental groups. Investigators performing the injections and survival monitoring were blinded to the group assignments. Humane endpoints were applied: mice were euthanized if they exhibited severe signs of distress, including hunched posture, ruffled fur, reduced mobility, or inability to reach food/water. All efforts were made to minimize animal suffering.

*S. aureus* strains (N315 WT, N315Δ*floA* and N315Δ*floA*::*floA*) were cultured to exponential phase. Bacterial suspensions were harvested, washed three times with PBS, and resuspended at a final concentration of 3 × 10^9^ CFU/mL. For survival experiments, cohorts of 8 mice were intraperitoneally injected with 0.1 mL of *S. aureus* suspension (3 × 10^9^ CFU/mL). Each strain was used to infect one mouse cohort, and a separate cohort was injected with an equal volume of sterile PBS as the negative control, followed by continuous monitoring and survival recording for 6 h post-infection. For inflammatory cytokine detection experiments, cohorts of 8 mice were intraperitoneally injected with *S. aureus* suspension (3 × 10^9^ CFU/mL) or sterile PBS (control). Six hours post-infection, blood was collected to isolate plasma. Then, the levels of tumor necrosis factor-α (TNF-α), interleukin-6 (IL-6), and interleukin-1β (IL-1β) were determined using an ELISA kit according to the manufacturer’s instructions (Jianglai Biotechnology Co., Ltd., Shanghai, China).

### 2.11. Statistical Analysis

All experiments were performed with three biological replicates. Statistical analyses were conducted using GraphPad Prism 10.0. Tables were visualized with Microsoft Word^®^ or Excel^®^. Data are expressed as the mean  ±  standard deviation (SD). One-way analysis of variance (ANOVA) was used for multi-group comparisons. Two-way ANOVA was performed to compare the differences among different strains under different temperature conditions. Survival curves were constructed using the Kaplan–Meier method. A *p* value < 0.05 indicated a significant difference, a *p* value < 0.01 indicated a highly significant difference, and a *p* value < 0.001 indicated a very highly significant difference.

## 3. Results

### 3.1. FMMs Contribute to the Hemolytic Activity of MRSA Without Affecting Bacterial Growth

**Figure 1:**
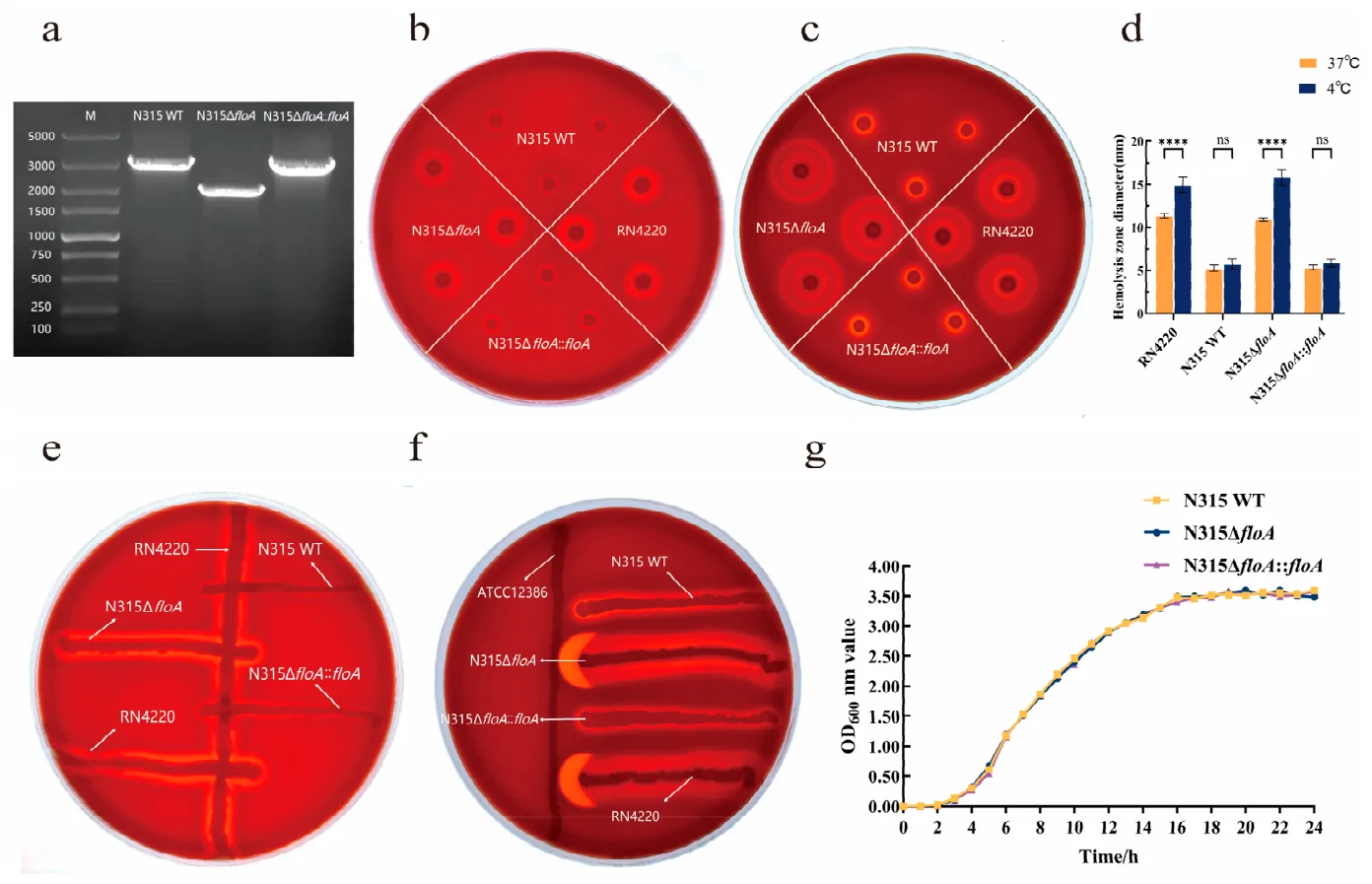
Disruption of FMMs enhanced the hemolytic activity of *S. aureus* N315: (**a**) Construction and identification of the FMM-disrupted strain and its complemented strain. (**b**) Columbia sheep blood agar plates were inoculated with N315 WT, N315Δ*floA*, N315Δ*floA*::*floA*, and RN4220 cultured at 37 °C overnight, and photographed. (**c**) Columbia sheep blood agar plates were further incubated with N315 WT, N315Δ*floA*, N315Δ*floA*::*floA*, and RN4220 cultured at 4 °C overnight, and photographed. (**d**) Evaluation of relative hemolysis zone diameters using *Adobe Photoshop* software. Statistical significance was calculated by two-way ANOVA. **** *p* < 0.0001, and “ns” represents no significance. (**e**) The cross-streaking tests for N315 WT, N315Δ*floA*, N315Δ*floA*::*floA*, and RN4220. Strains were tested against RN4220 to evaluate their hemolytic activities: β-hemolysin phenotype occurred in RN4220 and N315Δ*floA*, while neither N315 WT nor N315Δ*floA*::*floA* showed detectable hemolytic activity. (**f**) The CAMP test, using *S. agalactiae* ATCC12386 as the indicator strain; the test strains were streaked vertically on Columbia sheep blood agar plates and incubated at 37 °C for 24 h. The arrow-shaped hemolytic zone is characteristic of β-hemolysin positivity. (**g**) Growth curves of N315 WT, N315Δ*floA*, and N315Δ*floA*::*floA*. The schematic diagram shows the OD_600_ values of the target strains measured at the indicated time points. Data represent the mean of three independent replicates.

To investigate the effect of FMMs on the hemolytic activity of MRSA, FMM-disrupted and -complemented strains of the MRSA N315 strain were constructed. PCR electrophoresis results showed that the FMM-disrupted strain (N315Δ*floA*) lacked the approximately 990 bp *floA* fragment compared with N315 WT, and this deletion was further confirmed by Sanger sequencing. The band size of the FMM-complemented strain (N315Δ*floA*::*floA*) was consistent with that of N315 WT, and Sanger sequencing verified the correct integration of the *floA* gene without any mutations, confirming successful strain construction (Figure 1a). Hemolytic activity serves as a key virulence trait of *S. aureus*, and is closely related to its pathogenicity [38]. In this study, *S. aureus* RN4220, which only expresses β-hemolysin and has characteristic hot and cold hemolysis properties, was used as a positive control to investigate the regulatory effect of FMMs on the hemolytic activity of N315. After incubation on Columbia sheep blood agar plates at 37 °C for 12 h, the hemolysis zone diameter of N315Δ*floA* was significantly larger than that of N315 WT, and this phenotype was restored in N315Δ*floA*::*floA* (Figure 1b). After incubation at 4 °C for 12 h, the hemolysis zones of N315Δ*floA* and the RN4220 positive control were both significantly enlarged, displaying typical β-hemolysin cold-hot hemolysis characteristics (Figure 1c). Quantitative analysis showed that the hemolytic zone diameter of N315Δ*floA* at 4 °C was significantly increased by 4.95 mm compared with that at 37 °C (*p* < 0.05), and this phenotypic trend was consistent with that of RN4220. In contrast, the hemolytic zone diameters of N315 WT and N315Δ*floA*::*floA* only changed by 0.17 mm and 0.49 mm, respectively, between 4 °C and 37 °C, with no statistically significant difference (*p* = 0.3280, *p* = 0.2284) (Figure 1d).

Cross-streaking test results showed that RN4220 only expresses β-hemolysin; N315Δ*floA* exhibited a β-hemolysin phenotype, while neither N315 WT nor N315Δ*floA*::*floA* showed detectable hemolytic activity (Figure 1e). CAMP tests showed that both N315Δ*floA* and the positive control RN4220 formed characteristic arrow-shaped hemolytic zones at the inoculation line junction with *S. agalactiae* ATCC12386, whereas N315 WT and N315Δ*floA*::*floA* did not display this characteristic hemolytic zone (Figure 1f). To eliminate the potential impact of differences in bacterial growth on the hemolytic phenotype, we compared the growth rates of N315 WT, N315Δ*floA*, and N315Δ*floA*::*floA* and found no significant difference (Figure 1g), thereby ruling out growth-related effects on the hemolytic activity assay results.

### 3.2. FMMs Affect the Transcription of Hemolysins and Their Regulators in MRSA

**Figure 2:**
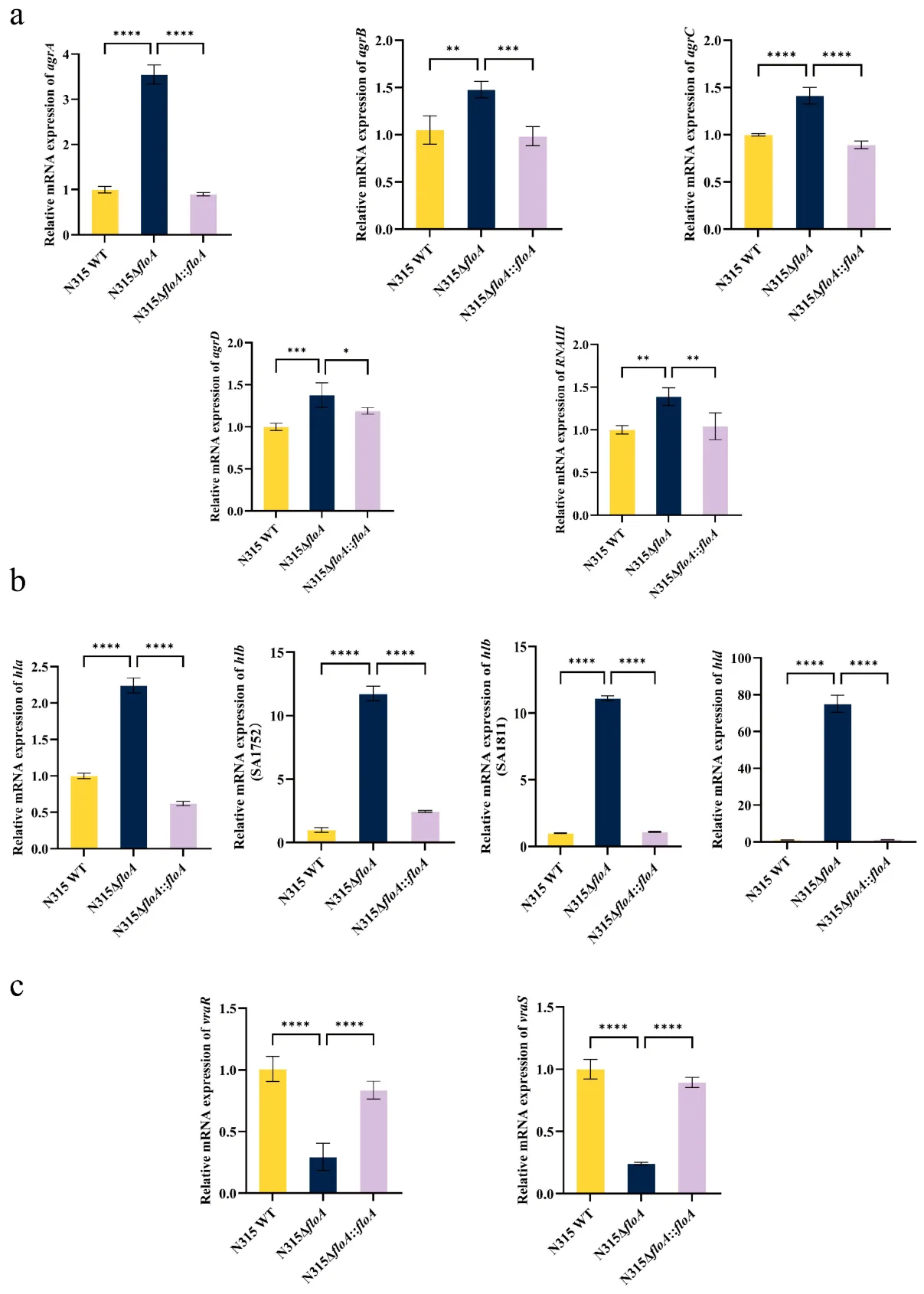
Quantitative real-time PCR analysis of the transcript levels of key virulence and regulatory genes among N315 WT, N315Δ*floA*, and N315Δ*floA*::*floA*. Results are presented relative to N315 WT; the corresponding expression value of N315 WT was normalized to 1. (**a**) Transcript levels of the co-transcribed agr operon (*agrB*, *agrD*, *agrC*, *agrA*) and its effector molecule RNAIII. (**b**) Transcript levels of hemolysin genes (*hla*, *hlb*-SA1752, *hlb*-SA1811, *hld*). (**c**) Transcript levels of the two-component system VraS/R (*vraS*, *vraR*). Bars represent mean values from three or more independent experiments. Error bars indicate standard deviations. **** *p* < 0.0001, *** *p* < 0.001, ** *p* < 0.01, * *p* < 0.05.

RT-qPCR analysis revealed that, compared with N315 WT, N315Δ*floA* exhibited significantly upregulated transcript levels of the co-transcribed agr operon (*agrB*, *agrD*, *agrC*, *agrA*) and its effector molecule RNAIII (Figure 2a), as well as the hemolysin genes *hla*, *hlb* and *hld* (Figure 2b). Notably, both truncated fragments of *hlb* (SA1752 and SA1811) were significantly upregulated in N315Δ*floA* (Figure 2b). In contrast, the transcript levels of *vraS* and *vraR* were significantly downregulated (Figure 2c). N315Δ*floA*::*floA* restored the expression of all these genes to levels comparable to those of N315 WT.

### 3.3. Disruption of FMMs Enhances the In Vivo Virulence of MRSA

**Figure 3:**
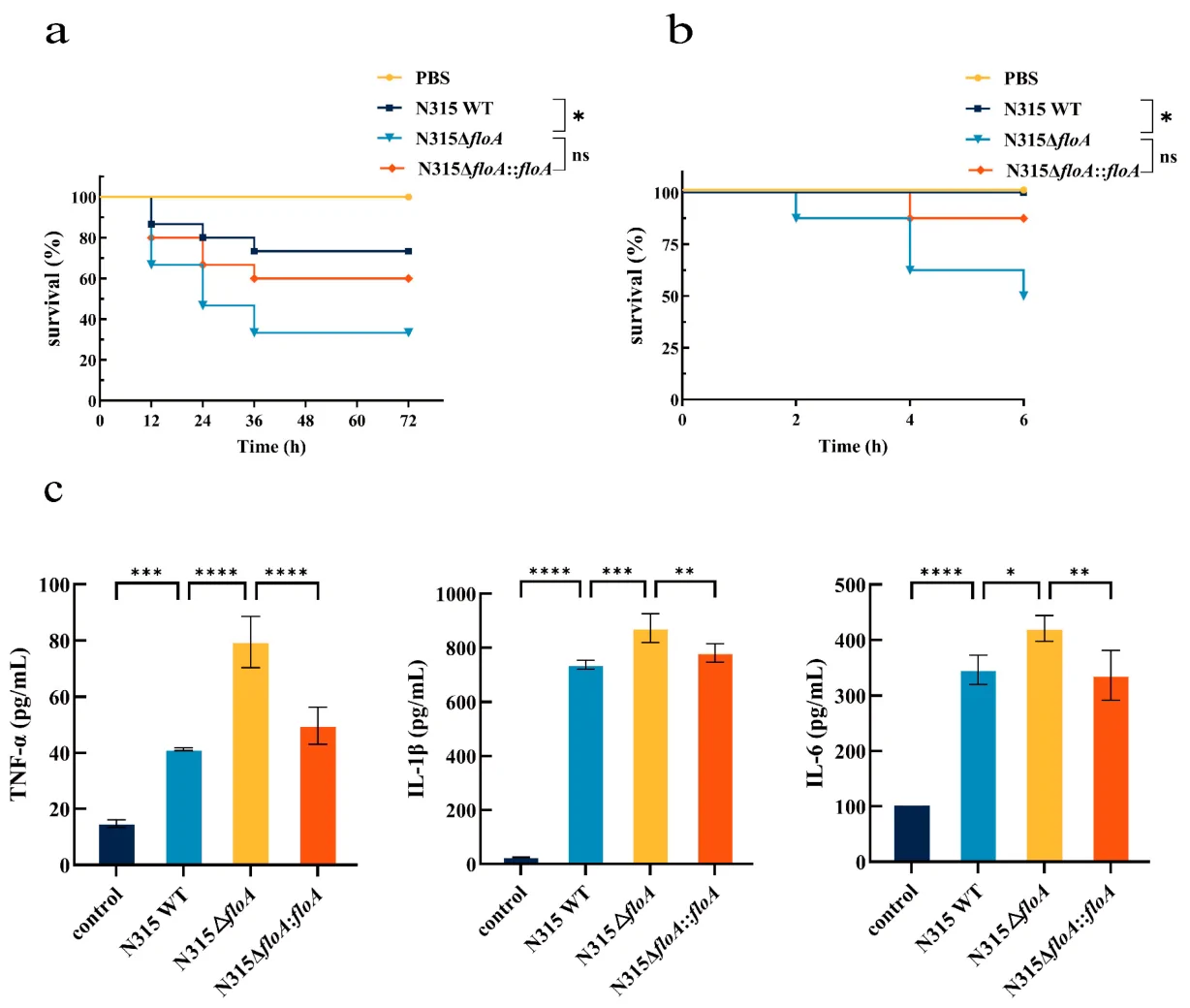
The effect of FMM disruption on the virulence of *S. aureus* N315 in vivo. (**a**) Survival analysis of *G. mellonella*. Each larva was injected with 10 μL of bacterial suspension (1 × 10^8^ CFU/mL) of N315 WT, N315Δ*floA*, or N315Δ*floA*::*floA*. The PBS-injected group served as a control. Larvae were incubated at 37 °C for 72 h, and survival was recorded at the indicated time points. Survival curves were generated using the Kaplan–Meier method, and statistical significance was determined by the log-rank test. * *p* < 0.05, and “ns” represents no significance. (**b**) Survival rates of mice infected with *S. aureus* (N315 WT, N315Δ*floA*, or N315Δ*floA*::*floA*). Mice were challenged via intraperitoneal injection with 0.1 mL of bacterial suspension containing 3 × 10^9^ CFU. Survival curves were constructed using the Kaplan–Meier method, and statistical significance was determined by the log-rank test. * *p* < 0.05, and “ns” represents no significance. (**c**) Detection of plasma pro-inflammatory cytokine levels after intraperitoneal infection. Six hours post-infection, plasma was collected from mice in N315 WT, N315Δ*floA*, N315Δ*floA*::*floA*, and PBS control groups. Levels of TNF-α, IL-1β, and IL-6 were measured by ELISA. Statistical significance was calculated by one-way ANOVA. * *p* < 0.05, ** *p* < 0.01, *** *p* < 0.001 and **** *p* < 0.0001.

To investigate the effect of FMMs on the in vivo virulence of *S. aureus*, invertebrate (*G. mellonella* larvae) and vertebrate (BALB/c mice) infection models were established. In the *G. mellonella* infection model, the PBS group showed a 100% survival rate. N315 WT group had a survival rate of approximately 73.3%, the N315Δ*floA* group had a survival rate reduced to 33.3%, while the N315Δ*floA*::*floA* group recovered to 60.0% (Figure 3a).

Similarly, in the BALB/c mouse intraperitoneal infection model, the PBS group had a 100% survival rate, the N315 WT group had a 100% survival rate, the N315Δ*floA* group survival rate dropped to 50.0%, while the N315Δ*floA*::*floA* group survival rate recovered to 87.5% (Figure 3b). The results of inflammatory factor testing showed that the PBS group had very low levels of inflammatory factors. Compared with the N315 WT group, mice in the N315Δ*floA* group had significantly elevated TNF-α, IL-6, and IL-1β levels, while the N315Δ*floA*::*floA* group had levels of all three inflammatory factors restored to wild-type levels (Figure 3c).

## 4. Discussion

*S. aureus* is one of the main pathogens causing hospital-acquired and community-acquired infections, and it can lead to a range of life-threatening infectious diseases, from skin and soft tissue infections to pneumonia and endocarditis [39]. The pathogenicity of *S. aureus* depends on the precise regulation of virulence gene expression by a multi-level regulatory network. The coordinated expression of various hemolysins, including α-hemolysin, β-hemolysin, γ-hemolysin, and δ-hemolysin, is the key virulence factor mediating host cell damage and the progression of infection [30,31]. Previous studies have confirmed that the Agr system may be negatively regulated by the two-component system VraS/R, which mediates the expression of virulence factors such as hemolysins through cross-regulation with the Agr system, thereby determining the pathogenic potential of the strain [17,18,19]. FMMs serve as a protein enrichment platform involved in the regulation of diverse biological processes including bacterial virulence, antibiotic resistance, biofilm formation, motility, and membrane homeostasis [25,27], whereas their regulatory role in hemolysins remains unclear. Currently, direct evidence is lacking to clarify whether FMMs modulate hemolytic activity and virulence factor expression in MRSA by regulating the canonical VraS/R-Agr signaling axis.

Hemolysin is one of the most important virulence factors of *S. aureus*, and its activity can be directly detected using blood agar plates [40,41]. Among them, β-hemolysin exhibits typical hot-cold hemolysis characteristics, and its high-level expression can significantly enhance the bacterium’s hemolytic activity and tissue-damaging ability [32,41]. In this study, the hemolytic phenotype of N315Δ*floA* was significantly enhanced on Columbia blood agar plates, with hemolytic activity close to that of the β-hemolysin-positive control strain RN4220. This phenotypic alteration was obviously restored in N315Δ*floA*::*floA*. Consistent with this finding, the cross-streak assay showed no obvious synergistic hemolysis between N315Δ*floA* and RN4220, further indicating that the enhanced hemolytic phenotype of N315Δ*floA* was primarily mediated by β-hemolysin. The CAMP assay revealed that N315Δ*floA* formed a typical arrowhead-shaped synergistic hemolytic zone with *S*. *agalactiae* ATCC 2386, whereas no obvious synergistic hemolysis was observed in N315 WT and N315Δ*floA*::*floA*. Meanwhile, there was no significant difference in the growth rates among all tested strains, ruling out the influence of growth status on hemolytic phenotypes. These results further confirm that FMMs negatively regulate β-hemolysin production in *S. aureus*. FMMs are crucial for maintaining the structural and functional integrity of cells, ensuring cellular homeostasis and signal transduction [42]. We speculate that disruption of FMMs may perturb the balance of membrane-mediated virulence regulatory pathways, thereby relieving the inhibition on the expression or secretion of β-hemolysin, and ultimately leading to enhanced hemolytic activity. This study not only reveals a novel role of FMMs in the regulation of β-hemolysin in *S. aureus*. In the N315 strain used in this study, the *hlb* locus is interrupted by the N315 prophage insertion, generating two separate fragments (SA1752 and SA1811). Despite this genomic configuration, our hemolysis assays revealed that N315Δ*floA* exhibited typical hot-cold hemolysis characteristic of β-hemolysin (Figure 1c,d), and both CAMP and cross-streaking tests confirmed the presence of β-hemolysin activity (Figure 1e,f). These findings suggest that prophage excision (a process known as phage-mediated *hlb* conversion) may occur in the N315Δ*floA* background, restoring an intact, functional *hlb* gene. Alternatively, the increased transcription of the truncated *hlb* fragments may contribute to the observed hemolytic phenotype through mechanisms that warrant further investigation. Future studies should examine whether FMM disruption influences prophage excision efficiency. It further enriches the regulatory network of virulence in *S. aureus* and offers a potential theoretical basis for the development of anti-virulence strategies targeting FMMs.

To further clarify the potential molecular mechanism by which FMMs regulate hemolysin expression, the transcriptional levels of virulence regulatory genes and hemolysin-encoding genes were determined by RT-qPCR in this study. Compared with N315 WT, the transcription levels of the co-transcribed agr operon (*agrB*, *agrD*, *agrC*, *agrA*) and its effector molecule RNAIII were significantly upregulated in N315Δ*floA*. Meanwhile, the transcription levels of hemolysin-related genes *hla*, *hlb*, and *hld* were also synchronously increased. In N315Δ*floA*::*floA*, the transcription levels of the above genes were significantly decreased relative to N315Δ*floA* and returned close to the levels of the N315 WT. Previous studies have demonstrated that the Agr system is a central virulence regulatory system in *S. aureus*, and its effector molecule RNAIII positively regulates the expression of *hla*, *hlb*, and *hld* [17,43]_._ These results suggest that the upregulation of hemolysin-related genes upon FMM disruption may be associated with the activation of the Agr-RNAIII regulatory axis.

In addition, the current study found that the transcription levels of the two-component system VraS/R genes (*vraS* and *vraR*) were significantly downregulated in N315Δ*floA*. Dai et al. [17] confirmed that VraR can directly bind to the promoter region of the agr operon, markedly repress the transcription of the Agr system, and subsequently downregulate the expression of RNAIII and downstream hemolysin-encoding genes, ultimately reducing the hemolytic virulence of the strain. Combined with the previous literature and our present findings, it is indicated that FMM disruption downregulates the transcription of *vraS* and *vraR*, which weakens the inhibitory effect of VraS/R on the Agr system. This leads to a significant increase in the transcription levels of RNAIII and downstream hemolysin-related genes (*hla*, *hlb*, *hld*).

As a protein-enriched membrane platform, FMMs play an essential role in the organization and stabilization of membrane protein complexes [27]. FMM disruption can disturb membrane protein localization and signal transduction [44]. Esther et al. [22] reported that VraS is localized to FMMs, and its function relies on the integrity of these membrane microdomains. Therefore, FMM disruption may impair the localization and function of VraS, thereby upregulating the transcription of downstream hemolysin-encoding genes via the VraS/R-Agr-RNAIII regulatory axis and eventually enhancing hemolytic virulence. Nevertheless, whether the VraS/R-Agr-RNAIII axis is involved in the regulation of hemolysin expression and virulence requires further verification in subsequent studies.

Given the critical role of hemolysins in disrupting the host immune barrier and inducing inflammatory injury [40,45], this study employed the *G. mellonella* larval infection model to evaluate the in vivo effects of disrupting FMMs. This model is cost-effective, ethically feasible, and possesses innate immune response that is conserved with mammals [46]. The results showed that after N315Δ*floA* infected *G. mellonella* larvae, the survival rate decreased significantly, confirming that the virulence of this strain in vivo is markedly enhanced, while N315Δ*floA*::*floA* can effectively reverse this virulence phenotype. Systemic infections caused by *S. aureus* pose a serious threat to host health and are typically accompanied by massive neutrophil migration and tissue infiltration [47,48]. Therefore, this study further established an acute intraperitoneal infection model in mice. The survival rate of mice infected with N315Δ*floA* was significantly lower compared to those infected with N315 WT and N315Δ*floA*::*floA*. Meanwhile, plasma pro-inflammatory cytokine detection results indicated that levels of TNF-α, IL-1β, and IL-6 in the plasma of mice infected with N315Δ*floA* were significantly upregulated, while N315Δ*floA*::*floA* restored them to levels similar to those of N315 WT.

**Figure 4:**
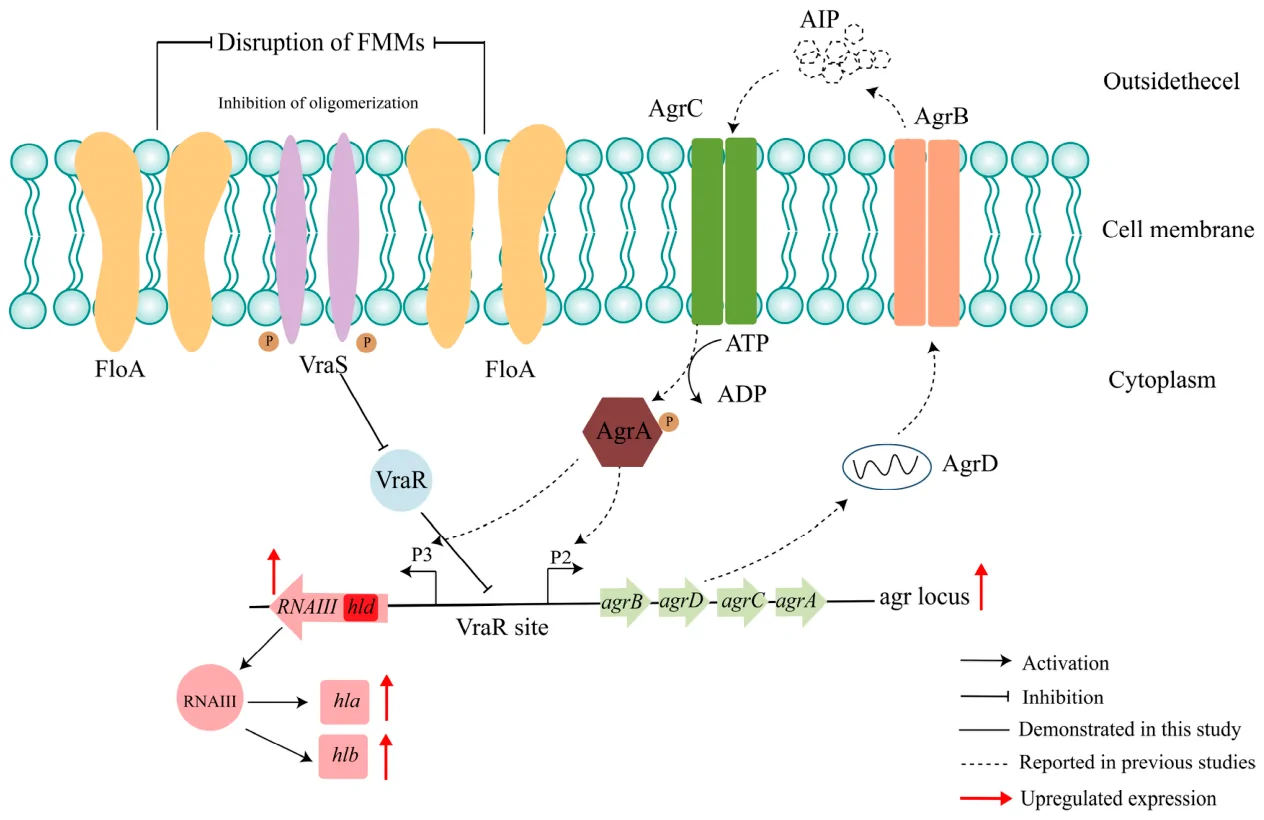
Schematic diagram showing the effect of FMMs on hemolysin expression in MRSA. Based on the experimental data of this study and previous reports, we propose that FMM disruption inhibits VraS oligomerization, thereby impairing VraR phosphorylation and its subsequent binding to the agr promoter. Loss of VraR-mediated repression of the agr locus activates the Agr system, upregulates RNAIII expression, and ultimately significantly enhances the expression of hemolysin genes (*hla*, *hlb*, and *hld*).

In this study, we demonstrate that FMMs function as a negative regulator of hemolysin production in *S. aureus* and attenuate bacterial virulence, likely through modulation of the VraS/R-Agr signaling axis (Figure 4). Thus, FMMs may ultimately affect the in vivo pathogenicity of this bacterium by regulating hemolysin expression and the host inflammatory response. Future work employing genetic dissection and mechanistic assays (e.g., electrophoretic mobility shift assay, Western blot, and RT-qPCR under defined conditions) will delineate how FMMs interface with VraS/R signaling and Agr regulation.

## 5. Conclusions

FMMs are negative regulators of hemolytic virulence of *S. aureus*. Disruption of FMMs enhances hemolysin gene transcription (*hla*, *hlb*, and *hld*) and thereby increases in vivo virulence, likely through modulation of the VraS/R-Agr signaling axis, which reveals a regulatory link between FMMs and hemolysin expression, enriching our understanding of MRSA virulence regulation. While FMM disruption enhances virulence, the opposite strategy—stabilizing or enhancing FMM function—could potentially suppress hemolysin expression and attenuate pathogenicity. This is conceptually analogous to anti-virulence approaches that target quorum-sensing systems to repress toxin production without exerting direct bactericidal pressure, thereby reducing the risk of resistance development. Thus, FMMs represent a promising target for anti-virulence strategies against MRSA infections, and further mechanistic dissection of this pathway may facilitate the development of novel therapeutic agents that modulate FMM integrity.

We acknowledge several limitations: all experiments were performed using a single MRSA strain (N315), and hemolysin changes were assessed at the transcriptional rather than protein level. Future work should validate these findings in additional clinical MRSA isolates, employ Western blot or proteomic approaches to quantify hemolysin protein production, and investigate whether pharmacological modulation of FMM integrity can serve as a viable anti-virulence strategy.

## Figures and Tables

**Table 1:** Strains and plasmids used in this study.

Strains/Plasmids	Description	Source
*Staphylococcus aureus*		
N315	a multidrug-resistant strain extensively used in *S. aureus* animal models	A kind gift from Prof. Xiancai Rao (Army Medical University, China) [29]
N315Δ*floA*	the FMM-disrupted strain with deletion of the *floA* gene in N315	This study
N315Δ*floA*::*floA*	The FMM-complemented strain generated by integrating pLI50-*floA* into N315Δ*floA*	This study
RN4220	an initial recipient for plasmid modification, which is introduced into *S. aureus* from *E. coli*	A kind gift from Prof. Xiancai Rao (Army Medical University, China) [29]
*Escherichia coli*		
DH5α	DNA cloning host strain	Weidi Bio, Shanghai, China
*Streptococcus agalactiae*		
ATCC12386	Indicator strain for CAMP test	Preserved in this laboratory
Plasmids		
pBT2	*E. coli-S. aureus* shuttle vector used for allelic exchange, Amp^R^, Cm^R^	A kind gift from Prof. Xiancai Rao (Army Medical University, China) [29]
pBT2-Δ*floA*	pBT2 recombinant plasmid carrying the upstream and downstream homologous arms of the *floA* gene, used for the construction of the FMM-disrupted N315 strain	This study
pLI50	*E. coli-S. aureus* shuttle cloning vector, Amp^R^, Cm^R^	A kind gift from Prof. Xiancai Rao (Army Medical University, China) [29]
pLI50-*floA*	pLI50 recombinant plasmid carrying the wild-type *floA* gene from N315	This study

Note: Cm^R^: chloramphenicol resistance; Amp^R^: ampicillin resistance. The antibiotics were used at the following concentrations: ampicillin at 100 μg/mL and chloramphenicol at 20 μg/mL for plasmid resistance.

**Table 2:** Primers used in this study.

Primers	Sequence (5′ → 3′)	Notes
*floA*-F	GACTCCCTCAACACGAA	Identification of the FMM-disrupted strain and its complemented strain
*floA*-R	GGATGACAACATCGAAAC	
*hla*-F	AGCGAAGTCTGGTGAAAACC	RT-qPCR
*hla*-R	TCTTGGAACCCGGTATATGGC	
*hlb*-SA1752-F	GTGCCAAAGCCGAATCTAAGAAAG	RT-qPCR
*hlb*-SA1752-R	GTCTCCAGTTTGGATACAAAACGG	
*hlb*-SA1811-F	CTCGGTCGTTCTCAATCAGGTTG	RT-qPCR
*hlb*-SA1811-R	CAATCGCTACGCCACCATCTTC	
*hld*-F	GGAAGGAGTGATTTCAATGGCA	RT-qPCR
*hld*-R	TGTTCACTGTGTCGATAATCCA	
*agrA*-F	GCGAAGACGATCCAAAACAAAGAG	RT-qPCR
*agrA*-R	GCGAGGGCAATTTCCATAGGC	
*agrB*-F	TGACCAGTTTGCCACGTATCTTC	RT-qPCR
*agrB*-R	ATTTGCATCCCTAAACGTACTTGC	
*agrC*-F	GATGACCCTATCATTCGCGTTGC	RT-qPCR
*agrC*-R	AGACCTAAACCACGACCTTCACC	
*agrD*-F	GCAATCGGAATTGTCGGTGG	RT-qPCR
*agrD*-R	AAATTCGTTAATTCAGCGGGTACT	
RNAIII-F	ACCGGCACATCATCGTCTATTCAC	RT-qPCR
RNAIII-R	GCACTTTCAGCAGCACGTTGTTC	
*vraS*-F	ACTGTTTCGCCTTCCATTGAACG	RT-qPCR
*vraS*-R	ATTGTTGGTTCGGTACTCGCATAC	
*vraR*-F	GAGTCGTCGCTTCTACACCATCC	RT-qPCR
*vraR*-R	ATTGCCAAAGCCCATGAGTTGAAG	
*hu*-F	GCTGGAACTTTACTTGCTGGGA	RT-qPCR
*hu*-R	TTGAGGTACGTGAACGTGCTG	

## Data Availability

The original contributions presented in this study are included in the article/supplementary materials. Further inquiries can be directed to the corresponding authors.

## Supplementary Materials

The following supporting information can be downloaded at: https://www.mdpi.com/article/10.3390/vetsci13080839/s1, Figure S1 is original image of Figure 1a.
