# Supplementary material for: Disruption of Functional Membrane Microdomains Enhances Methicillin-Resistant Staphylococcus aureus Pathogenesis via Hyperexpression of Hemolysins
Source: Vet Sci. 2026 Aug 20;13(8):839. doi: 10.3390/vetsci13080839 (PMC13517402; doi:10.3390/vetsci13080839)

Supplementary Uncropped Gels

Uncropped gel images are provided. The boxed area indicates the lanes shown in Fig. 1a; lanes outside the box correspond to samples not included in this figure.

Figure S1 Uncropped gel (original)

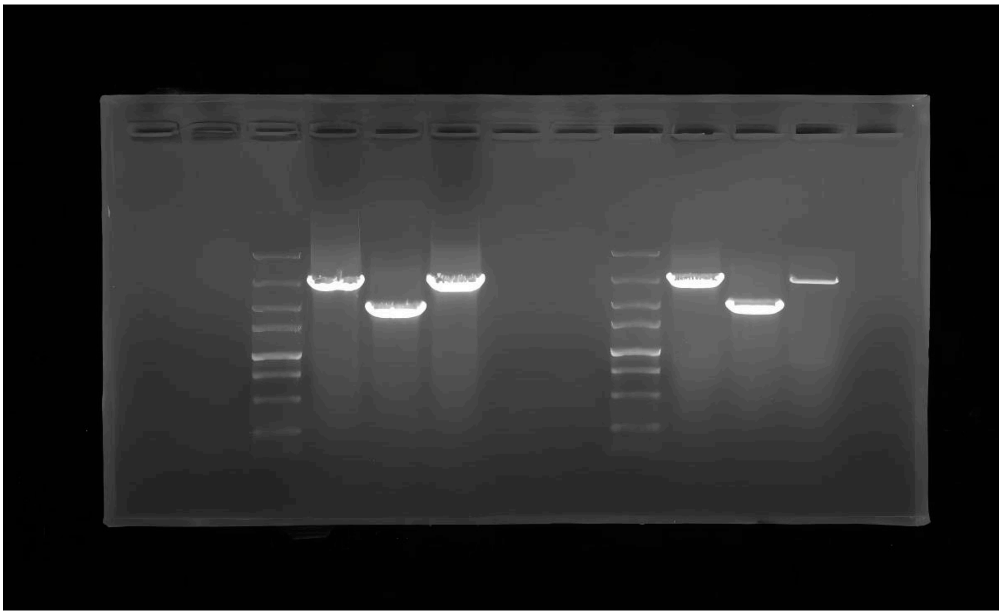

Annotated uncropped gel (boxed lanes shown in Fig. 1a)

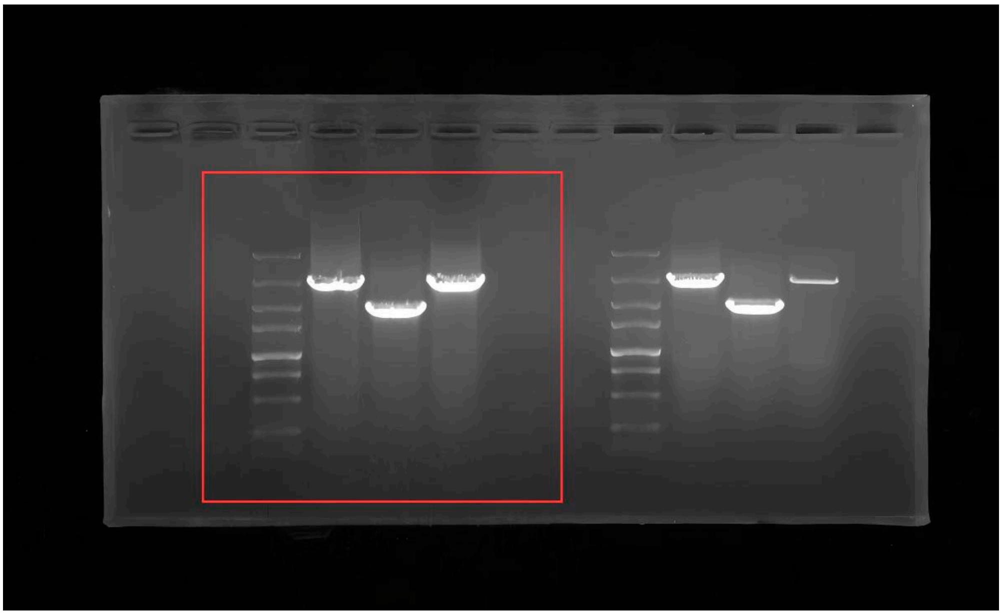

Supplement: Supplementary file 1 [file vetsci-13-00839-s001.zip › vetsci-4434126-supplementary.pdf]
